# From Zirconium Nanograins to Zirconia Nanoneedles

**DOI:** 10.1038/srep33282

**Published:** 2016-09-13

**Authors:** E. Zalnezhad, A. M. S. Hamouda, J. Jaworski, Young Do Kim

**Affiliations:** 1 Department of Mechanical Convergence Engineering, Hanyang University, 222 Wangsimni-ro, Seongdong-gu, Seoul, 04763, Korea; 2 Department of Mechanical and Industrial Engineering, Qatar University, Doha, Qatar; 3 Department of Chemical Engineering, Hanyang University, 222 Wangsimni-ro, Seongdong-gu, Seoul, 04763, Korea; 4 Department of Materials Science and Engineering, Hanyang University, Seoul 04763, Korea

## Abstract

Combinations of three simple techniques were utilized to gradually form zirconia nanoneedles from zirconium nanograins. First, a physical vapor deposition magnetron sputtering technique was used to deposit pure zirconium nanograins on top of a substrate. Second, an anodic oxidation was applied to fabricate zirconia nanotubular arrays. Finally, heat treatment was used at different annealing temperatures in order to change the structure and morphology from nanotubes to nanowires and subsequently to nanoneedles in the presence of argon gas. The size of the pure zirconium nanograins was estimated to be approximately 200–300 nm. ZrO_2_ nanotubular arrays with diameters of 70–120 nm were obtained. Both tetragonal and monoclinic ZrO_2_ were observed after annealing at 450 °C and 650 °C. Only a few tetragonal peaks appeared at 850 °C, while monoclinic ZrO_2_ was obtained at 900 °C and 950 °C. In assessing the biocompatibility of the ZrO_2_ surface, the human cell line MDA-MB-231 was found to attach and proliferate well on surfaces annealed at 850 °C and 450 °C; however, the amorphous ZrO_2_ surface, which was not heat treated, did not permit extensive cell growth, presumably due to remaining fluoride.

One-dimensional nanoscale materials such as nanoneedles, nanowires, nanoribbons, nanofibers, nanorods, and nanotubes have attracted interest lately, owing this to their significance in potential technology applications and basic scientific research. In particular, ZrO_2_ has been broadly explored, owing this to the variety of potential applications related to its good physical and chemical properties, including a high melting point, mechanical and thermal resistance, low electrical conductivity, chemical inertness, permeability, and biocompatibility. ZrO_2_ is widely employed in transparent optical components, electrochemical capacitors, membrane reactors, microelectronics, protective coatings, as components in oxygen sensors, magnetic materials, heterogeneous catalysts, as electrolytes in solid-oxide fuel cells, and as biomaterials in medical implants^1^,^2^. Furthermore, ZrO_2_ can also be utilized in environmental applications involving catalytic purification of harmful gases and in the synthesis of biodiesel^3^,^4^. The morphology and structure of ZrO_2_ have an important influence on its use. For example, the catalytic activity of zirconia nanosized-particles is enhanced because of their large specific surface area^5^. Nevertheless, separation of nanosized-particles from the reaction medium is difficult, and loss of a significant amount of the nanosized-particles during the recycle process may occur. Therefore, the enhancement of ZrO_2_ nanocatalysts require more investigation. ZrO_2_ nanotubes have recently been synthesized using an anodic oxidation technique^6^,^7^,^8^.

ZrO_2_ nanocrystalline powder^9^, thin films^10^, nanotubes^11^, nanorods^12^, nanobelts^13^, nanowires^14^, and nanoneedles^15^ can be synthesized using versatile methods. Zirconia nanotubes have been constructed using template-assisted deposition, hydrothermal treatment, and anodic oxidation techniques. Cao *et al*. reported the synthesis of tetragonal zirconia nanowires for optical applications using template methods^16^,^17^.

Control of the surface morphology and crystallinity is vital for numerous zirconia thin film applications. To enhance the sensitivity of film humidity sensors, a surface with a large surface area and high porosity is needed. There are many techniques for zirconia thin layer deposition, including physical vapor deposition (PVD), sputtering, chemical vapor deposition (CVD), electron-beam deposition, and plasma spraying. Unfortunately, these methods are rather expensive and it is hard to obtain highly porous layers^18^. Ksapabutr *et al*. reported on zirconium dioxide nanostructures layers with diverse morphologies deposited on a glass substrate through electrostatic spray deposition using zirconatrane as a precursor. In their work, the zirconia structures showed nanoneedle- and nanoflower-like morphologies. The humidity sensing properties of sensors based on these ZrO_2_ thin layers were examined. Flower shaped zirconia-based humidity sensors had better sensitivity, quicker recovery behavior, and higher reproducibility compared to sensors based on needle shaped zirconia^19^. Liu *et al*. reported the synthesis of ZrO_2_ nanorods by annealing of precursor powders prepared in a novel inverse microemulsion system^20^,^21^.

To our knowledge, no research has been done on the gradual synthesis of zirconium nanograins and their conversion to zirconia nanoneedles by a combination of PVD magnetron sputtering, anodization, and annealing in an argon ambient.

Plasma spraying is the preferred commercial coating technique for zirconia. However, plasma spraying has several significant disadvantages, including poor coating-to-substrate adhesion, the presence of different undesired phases, and low coating cohesion. As a result, a number of alternative techniques have been investigated to overcome the disadvantages of plasma spraying techniques including sol-gel, anodic oxidation, PVD, and CVD^1^,^22^. One of the methods is the application of PVD in order to create thin oxide Zr-, Ti- Si-, P-, and Ca-based layers^23^.

The goal of this study is to produce ZrO_2_ nanoneedles from pure zirconium (Zr) nanograins by simple procedures in which each product during each step of the procedure has its own application (e.g., biomedical applications, sensors, capacitors, magnetic materials, and energy).

In this study, a pure thin film zirconium (nanograins) was coated atop the substrate by PVD magnetron sputtering. The Zr coated substrates were anodized to fabricate ZrO_2_ nanotubes arrays. ZrO_2_ thin film coated specimens were annealed at different annealing temperatures, including 250, 450, 650, 850, 900, and 950 °C, in the presence of argon gas. X-ray diffraction (XRD) and field emission scanning electron microscopy (FESEM) were utilized for coating and structural characterization. The ability of the surfaces to afford cell attachment and permit cellular growth and proliferation were examined. In brief, a difference in the biocompatibilities was observed depending on heating of the ZrO_2_.

## Results

**Figure 1:**
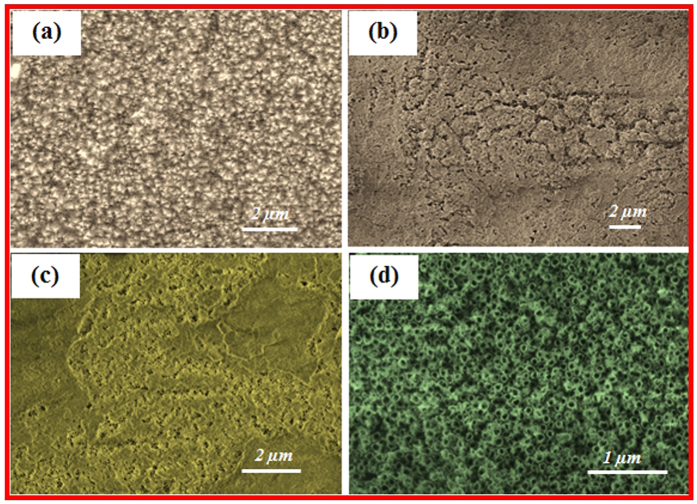
SEM images showing the top view of (**a**) Zr coated substrate and ZrO_2_ nanotubular array-coated substrate at an anodic oxidation duration of (**b**) 20 min, (**c**) 30 min, and (**d**) 60 min.

The primary surface of the zirconia coated substrate is fairly flat, and Zr nanograins with grain sizes of approximately 200–300 nm were deposited atop the substrate, as shown in Fig. 1a. There were apparent changes in the morphology of the surface throughout the anodic oxidation process. Figure 1b–d show the SEM images of the ZrO_2_ coated specimens after anodic oxidation in a mixture of ammonium fluoride electrolyte dissolved in a 95:5 glycerol and distilled water solvent mixture at ambient temperature. Figure 1b presents the structure of the surface of the nanotubes after 20 minutes. Figure 1c shows an SEM image of the anodized samples after 30 minutes. As shown, nanopores begin to form as a first step toward ZrO_2_ nanotube formation. When the time was increased to 60 minutes, ZrO_2_ nanotubes clearly formed atop the substrate (Fig. 1d). The surface was not flat and was partially covered by a loose solid substance. Ultrasonic washing after anodic oxidation separated most of the solids covering the nanotubes to reveal the nanotube structure (Fig. 1d).

Both the formation and dissolution of the ZrO_2_ layer happened concurrently. When anodic oxidation occurs, OH^−^ and F^−^ accumulate at the anode surface. The zirconium atom loses an electron and reacts with OH^−^ to form insoluble ZrO_2_. Additionally, Zr^4+^ can react with F^−^ to form [ZrF_6_]^2−^ and diffuse into the solution. After the combination of OH^−^ and F^−^ with Zr^4+^, OH^−^ and F^−^ in the solution migrated to the anode surface. The OH^−^ and F^−^ at the anode surface were replenished from the solution. Dense ZrO_2_ layers formed under these conditions^24^.

The potential applications and properties of ZrO_2_ hinge on its microstructures and crystallinity. In order to explore the crystallization transformation of the nanotube arrays, the amorphous ZrO_2_ coated substrates were heat treated in an argon gas furnace for 2 and 4 hours.

Based on the X-ray diffraction studies, we observed that all nanostructured layers prepared in this study had a crystalline structure. ZrO_2_ nanotubes, which have an amorphous structure, can only be used in applications that involve medical implants, catalysts and photocatalysts^25^. To convert the amorphous structure into a crystalline structure, it is essential to apply heat treatment, which was also used to eliminate the remaining fluoride.

**Figure 2:**
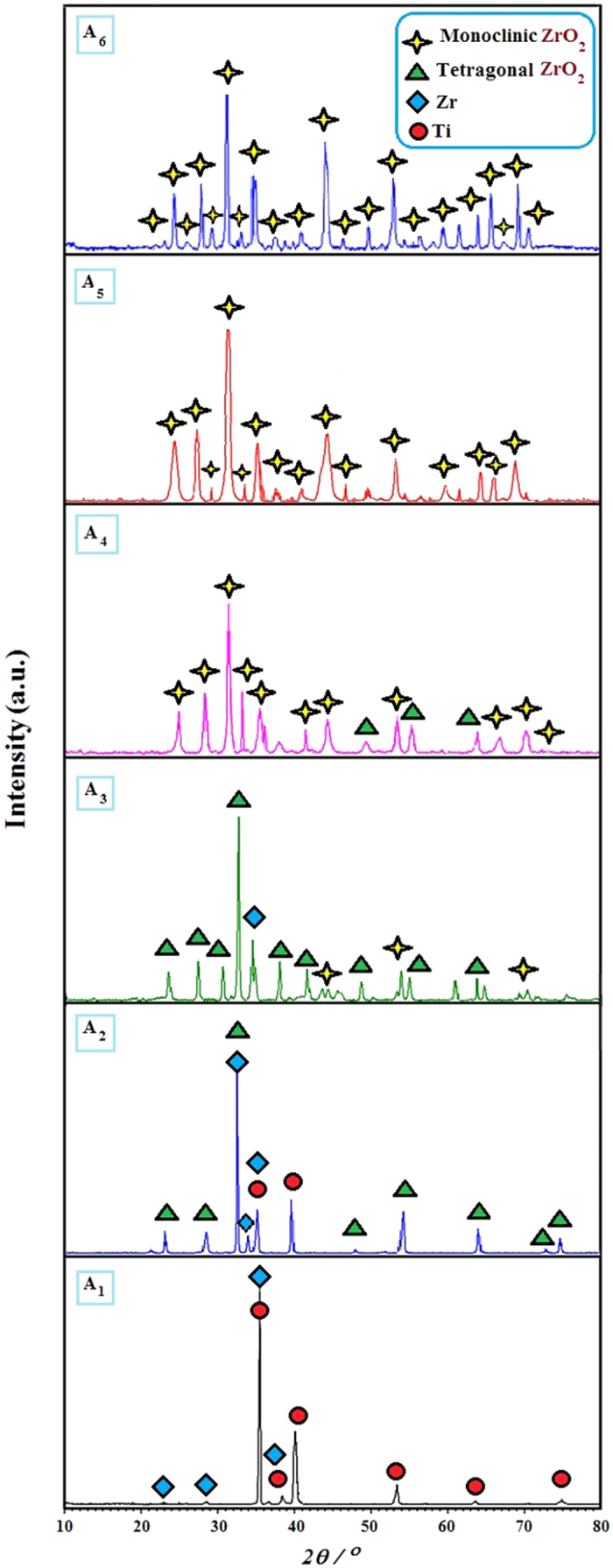
XRD spectra of Zr coated (A1) and ZrO2 coated substrates annealed at 450 °C (A2), 650 °C (A3), 850 °C (A4), 900 °C (A5), and 950 °C (A6).

Generally, there are three phases of zirconia: tetragonal, monoclinic, and cubic. The first two phases can usually be achieved at temperatures below 1000 °C^26^. In this study, we focused only on tetragonal and monoclinic phases. The XRD patterns of the Zr (nanograin) deposited specimens and the heat treated specimens at different annealing temperatures are shown in Fig. 2. XRD patterns of the Zr coated substrate and ZrO_2_ coated substrates at different annealing temperatures (450, 650, 850, 900, and 950 °C) are denoted inside the XRD graph by A_1_, A_2_, A_3_, A_4_, A_5_, and A_6_, respectively. The ZrO_2_ nanotube-coated substrates were amorphous before annealing. At an annealing temperature of 450 °C, most of the diffraction peaks can be indexed as the tetragonal phase, in which the main peak of tetragonal zirconia appeared at 2*θ* = 34°. At 650 °C, the intensity of the peaks for both the tetragonal (pdf Card No. 50–1089) and monoclinic phases were enhanced due to more complete crystallization. At 850 °C, the zirconia nanotube arrays were of a monoclinic phase (pdf Card No. 37–1484), and only a few diffraction peaks of tetragonal zirconia appeared. Further increases in annealing temperature to 900 and 950 °C resulted in an increase in the intensity of the monoclinic phase.

**Figure 3:**
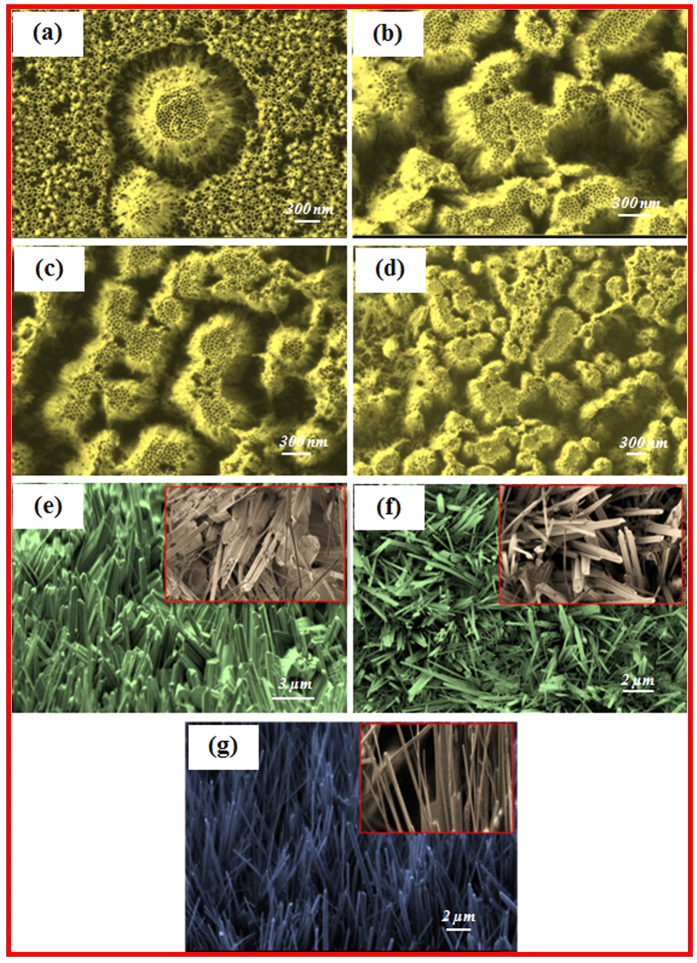
SEM image top views of the ZrO_2_ thin films: (**a**) nanotubes annealed at 250 °C, (**b**) 450 °C, (**c**) 650 °C, and (**d**) 850 °C. (**e**) Nanorods annealed at 900 °C for 2 hours and (**f**) nanorods and nanowires annealed at 900 °C for 4 hours. (**g**) Nanoneedles annealed at 950 °C for 2 hours.

Figure 3a–g illustrate the SEM top views of ZrO_2_ coated substrates at different annealing temperatures. Figure 3a shows SEM views of ZrO_2_ nanotubes annealed at 250 °C for 2 hours. As can be seen, two bunches of nanotubes shaped like a volcano between other nanotubes appeared after annealing. Further increases in temperature to 450, 650, and 850 °C led to the generation of more volcano-shaped bunches of nanotubes (Fig. 3b–d). These were observed after annealing for 2 hours. In all cases (250–850 °C), an increase in annealing time to 4 hours had no significant effect on the morphology of the ZrO_2_ nanotubes as compared to annealing for 2 hours.

Figure 3e presents ZrO_2_ thin films annealed at 900 °C for 2 hours. The morphology and structure of the nanotubes changed to nanorods. A magnified SEM is provided in the inset of Fig. 3e. As can be seen in this inset, the surfaces showed distributions of nanorods (major) and some nanowires (minor). For further investigation, we performed the annealing process at 900 °C for 4 hours to see the effect of both temperature and time. More nanowires and a few nanoneedles were generated under these conditions, as can be seen in the magnified SEM images inserted in Fig. 3f. Figure 3g shows the morphology of the ZrO_2_ after annealing at 950 °C for 2 hours. As shown, more nanoneedles and some nanowires were observed after annealing at 950 °C for 2 hours.

**Figure 4:**
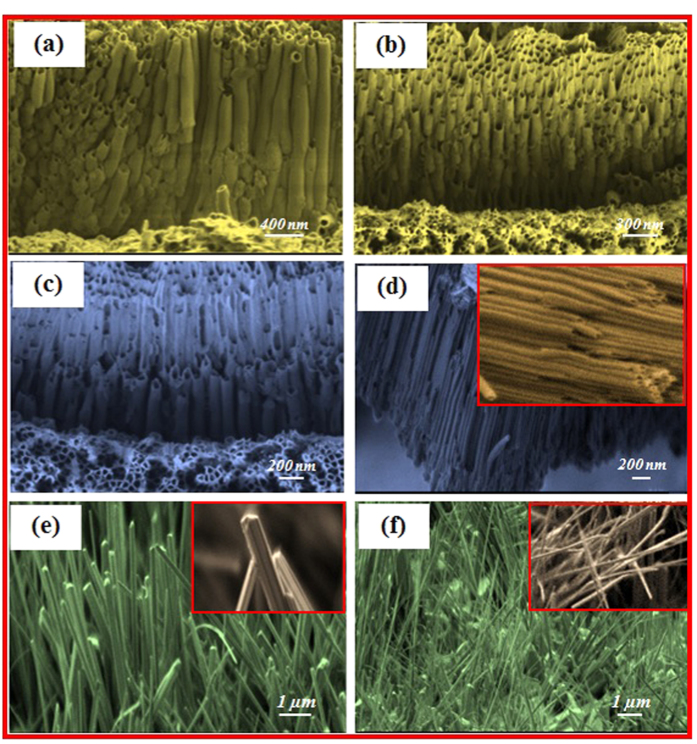
SEM image cross-section views of ZrO_2_: (**a**) nanotubes as-prepared and (**b**) annealed at 450 °C, (**c**) 650 °C, and (**d**) 850 °C. (**e**) Nanorods annealed at 900 °C for 4 hours and (**f**) nanoneedles annealed at 950 °C for 4 hours.

To further study the morphology and structure of ZrO_2_ nanotubes, nanorods, nanowires, and nanoneedles, cross sectional images were obtained before and after annealing. Figure 4a shows the cross-sectional images of unannealed ZrO_2_ nanotubular array coated substrates (as-prepared). Figure 4b shows the ZrO_2_ nanotubes after annealing at 450 °C. With further increases in annealing temperature to 650 °C, the nanotubes became more brittle due to the ceramic nature of ZrO_2_ (Fig. 4c). Figure 4d shows the ZrO_2_ nanotubes annealed at 850 °C; as shown, the tubes became much more compact. Figure 4e,f present the cross-sectional views of ZrO_2_ after annealing at 900 and 950 °C for 4 hours, respectively. As depicted, the morphology and structures of nanotubes changed to nanorods, nanowires and nanoneedles with diameters of 80–110 nm, 70–100 nm and lengths of 40–60 nm, respectively.

**Figure 5:**
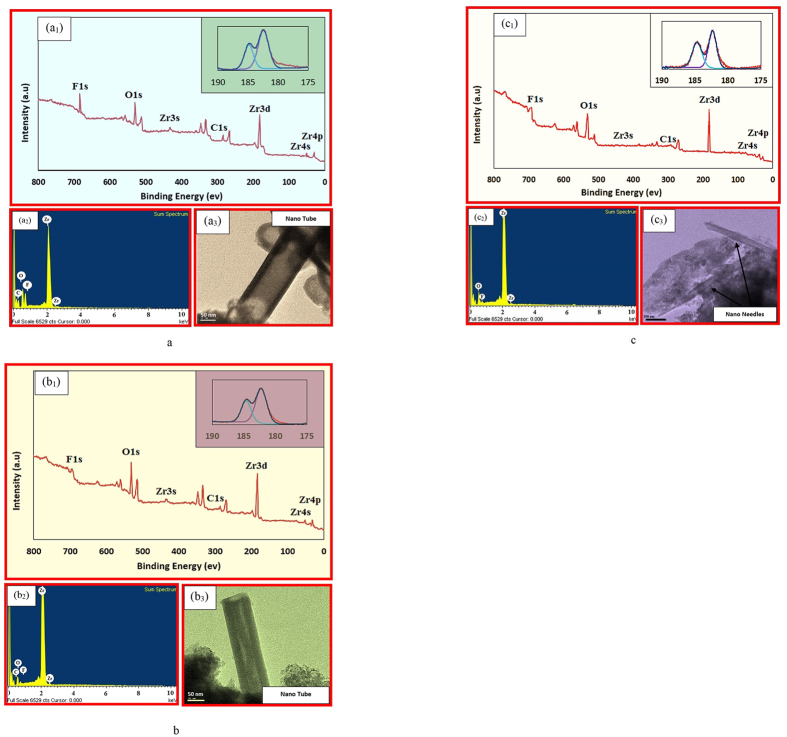
(**a**) XPS spectrum (a_1_), EDX analysis (a_2_), and TEM image (a_3_) of amorphous ZrO_2_. (**b**) XPS spectrum (b_1_), EDX analysis (b_2_), and TEM image (b_3_) of ZrO_2_ annealed at 450 °C. (**c)** XPS spectrum (c_1_), EDX analysis (c_2_), and TEM image (c_3_) of ZrO_2_ annealed at 950 °C.

XPS, EDX, and TEM images of ZrO_2_ coating before and after annealing at 450 °C for two hours and 950 °C for four hours present in Fig. 5a–c. XPS spectrum (a_1_), EDX analysis (a_2_), and TEM image (a_3_) of amorphous ZrO_2_, XPS spectrum (b_1_), EDX analysis (b_2_), and TEM image (b_3_) of ZrO_2_ annealed at 450 °C, and XPS spectrum (c_1_), EDX analysis (c_2_), and TEM image (c_3_) of ZrO_2_ annealed at 950 °C.

**Figure 6:**
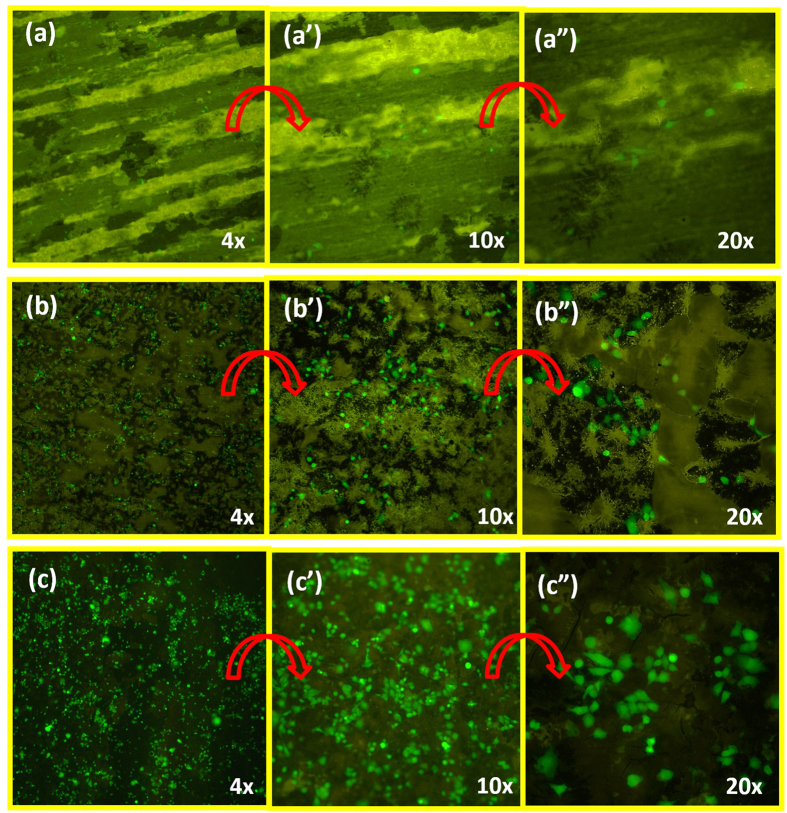
Biocompatibility of the ZrO_2_ coated substrate using the human cell line MDA-MB-231 possessing a green fluorescent protein as a reporter for living cells. Amorphous ZrO_2_ (**a–a**”), ZrO_2_ coated sample annealed at 450 °C (**b–b**”), and ZrO_2_ coated sample annealed at 850 °C (**c–c**”).

Finally, we evaluated the biocompatibility of the ZrO_2_ surface using the human cell line MDA-MB-231 possessing a green fluorescent protein as a reporter for living cells. As shown in Fig. 6, the exposure of the surfaces to heat treatment, specifically during annealing, significantly influenced the ability for the cells to attach and proliferate on the surface. The heat treated surfaces of ZrO_2_ at 450 °C and 850 °C displayed a high degree of cell attachment, spread, and proliferation as determined by the fluorescence and characteristic morphology of the green fluorescent protein (GFP) expressing adenocarcinoma cells. In contrast, a negligible number of cells could be observed on the non-annealed ZrO_2_ surface.

## Discussion

The ZrO_2_ nanotubes were annealed at different temperatures and the evolution of the cross-section morphologies during heat treatment in presence of argon gas were studied. The morphologies of ZrO_2_ nanotubes annealed at 250, 450, 650, and 850 °C maintained their original nanotubular form, but the surface became more compact gradually. With further increase in annealing to 900 °C, the cracks initiated in zirconia nanotubes and broke into particles. This phenomenon may be due to the stress concentration in ZrO_2_ ceramic nanotubes owing to heat treatment and propagation of cracks because of tubes’ wall expansion with further enhance in annealing temperatures. When ZrO_2_ annealed at 900 °C, the ZrO_2_ nanotubes developed into rods, and wires. Based on the experimental observations along with other researchers’ reports, it can be interpreted in the way that the nanotubes after annealing at 250, 450, 650, and 850 °C are gradually compacted and become more brittle due to its ceramics nature (see Fig. 3a–d). The particles of some nanotubes which are in contact with other tubes try to coalesce together during the annealing at higher temperature beyond 850 °C and due to tensional thermal stress concentration and residual stress, the cracks generate. After fracture, the tubes at the coalesced part with others may create nanorods, while, other parts of the wall of the tube may fracture to nanowires and nanoneedles. As can be seen form Figs 3 and 4, the nanorodes, and wires are separated, so, with further increase in temperature to 950 °C at 2 and 4 hr they cannot coalesce, instead, they fractured and converted to nanoneedles.

XRD patterns of the as-prepared ZrO_2_ nanotubes and the nanotubes annealed at different temperatures are shown in Fig. 2. The as-prepared sample is an amorphous structure. At 450 and 650 °C, most of the diffraction peaks are indexed as the tetragonal phase. At annealing temperatures 850, 900, and 950 °C, most of the diffraction peaks are indexed as the monoclinic phase. The transition from amorphous to crystalline, initially can be defined by the resemblance between the tetragonal phase and the amorphous structure. First *et al*.^26^ explained that the distribution function analysis for the tetragonal phase and the amorphous structure specifies that they may have the similar first-neighbor distance, which means the two phases have a same short-range order, therefore, the phase transformation from amorphous to tetragonal together with fewer distortion of structure may happen when temperature enhances from ambient temperature to 400 °C.

The presence of the three polymorphs of zirconia was also a function of the particle size. That is, as the crystallites decreased in size, a critical size was reached when the tetragonal form was more stable than the monoclinic form. At even finer grain sizes, the cubic form was the most stable form. An explanation for this is given in terms of the surface energy of the three polymorphs, which follows the trend *γ*_*mono*_ > *γ*_*tet*_ > *γ*_*Cube*_. So, as the total surface area increased, the monoclinic form became metastable relative to the tetragonal form. A microscopic examination of hardness-tested monoclinic ZrO_2_ shows (100) and (110) twinning on a scale as fine as 10 nm in two orthogonal directions. This provides multiaxial, nearly continuous plastic deformation and explains the unexpected softness of the monoclinic form^26^,^27^.

Figure 5a–c displays the XPS, EDX, and TEM images of ZrO_2_ amorphous, annealed at 450 °C and annealed at 950 °C, respectively. Figure 5a1 shows XPS spectra of the Zr3d and O1s core levels of amorphous ZrO_2_. The peak located at 182 eV are attributed to the Zr3d components. The binding energy of O1s in ZrO_2_ is located at 531 eV. The F1s peak was found to be at 684 eV. Clearly, in the as-prepared anodic ZrO_2_ nanotubes, there are significant amounts of fluorine species. It is clear from the XPS spectrum result that fluoride ions were embedded in the ZrO_2_ nanotubes owing to the competition of fluoride and oxygen ions during anodic oxidation^28^,^29^.

XPS spectrum of ZrO_2_ annealed at 450 °C and 950 °C are present in Fig. 5b1 and Fig. 5c1, respectively. The spectrum revealed dominant peaks of Zr, and O along with a C1s peak due to the presence of hydrocarbon species as surface contamination. The Zr3d peak has binding energies of 183 eV, which represents the fully oxidized zirconium ion in its Zr4+ state^30^,^31^. The O1s peak was found to be at 531 eV. After annealing at 450 °C, the nanotubes are still uniform (see Fig. 5b3), similar to the as-prepared samples, as shown in the TEM image in Fig. 5(b3). The EDX spectrum of ZrO_2_ nanotubes annealed at 450 °C, as shown in Fig. 5(b2), clearly reveals that the annealing treatment causes the elimination of fluorine species. After annealing at 450 °C, the Zr3d and O1s peaks were found to be at 182 and 530 eV, respectively, the nanotubes changed to nanoneedles (see Fig. 5c3).

Figure 5c_1-2_ presents EDX and TEM image of ZrO_2_ annealed at 950 °C. As can be seen from EDX the intensity of fluorine is close to zero (XPS spectrum result also confirms it, see Fig. 5c1). This is likely due to evaporation as HF or F2 gas species during the heat treatment. Another plausible reason could be the flexibility of the amorphous state. Incorporation of rather large amounts of additional impurities in amorphous solids is allowed by reduced structural constraints in the amorphous network. During crystallization processes foreign atoms simply pull off from the volume of growing crystallites. Figure 5c3 shows TEM images of ZrO_2_ nanoneedles after annealing at 950 °C for 4 hours.

In looking at the biocompatability, the non-annealed amorphous ZrO_2_ surface was not capable of providing extensive attachment and growth of the adenocarcinoma cells, as may be expected due to the remaining presence of fluoride on the surface. In stark distinction, the ZrO_2_ surfaces after annealing at 450 °C allowed cell attachment and proliferation as was observed by the bright fluorescent signal from the green fluorescent protein (GFP) produced within the viable MDA-MB-231 cells. In addition, the cell spreading on the surface was indicative of a characteristic epithelial morphology suggest strong adhesion and biocompatibility of the surface. The biocompatibility of the ZrO_2_ surface annealed at 850 °C similarly permitted cell spreading and proliferation.

## Methods

### Deposition of pure Zr layer

Substrates with 20 × 10 × 5 mm^3^ dimensions were cut from Ti-6Al-7Nb plates. The surfaces of the substrates were polished using silicon carbide emery paper with grits from 800 to 2500. Subsequently, the samples were polished with cloth paper to a mirrored finish. The substrates were ultrasonically sonicated in acetone and ethanol to eliminate organic contamination and were subsequently washed with deionized water and were dried at 100 °C for 14 min. Then, a TF450 physical vapor deposition magnetron sputtering apparatus (SG Control Engineering, Singapore) was utilized for coating a pure zirconium nanograin thin film atop the samples. A Zr target with 99.995% purity was utilized in this study. The coating machine has a vertical configuration in which two switchable RF and DC targets were placed above a rotatable substrate holder at a 150 mm distance. Before Zr deposition, the target was pre-sputtered in an argon atmosphere for 20 min to eliminate the oxide layer. Evacuation of the PVD chamber was performed at below 2 × 10^−5^ Torr prior to the insertion of argon gas for the deposition process. The pressure of the PVD chamber was kept at 5.2 × 10^−3^ Torr, and the flow rate of argon gas was maintained at 25 sccm during 3 hours of deposition. The PVD deposition parameters, including DC power, DC bias voltage, and temperature, were adjusted at 300 W, 75 V and 200 °C, respectively^32^.

### Anodic oxidation of Zr-coated substrate

The pure zirconium films on their substrates were anodized in a solution containing NH_4_F (Sigma-Aldrich CO., 0.5 wt%) electrolytes dissolved in a 95:5 glycerol (Sigma-Aldrich CO., 99 wt%) and distilled water solvent mixture at ambient temperature. Anodic oxidation was performed in an electrochemical cell with two electrodes in which a 5 mm graphite rod was selected as the counter electrode. The counter and working electrodes were separated at a distance of around 20 mm. The anodization was carried out using a direct current (DC) power supply (E3641A, Agilent Technologies, Palo Alto, USA) at 60 V for diverse anodization times of 20, 30, and 60 minutes. Heat treatment was performed using a furnace in the presence of argon gas^32^ at 250, 450, 650, 850, 900, and 950 °C, for 2 and 4 hours with heating and cooling rates of 3 °C min^−1^.

### Characterization

The structural morphology and elemental analysis of the coatings were characterized using a high-resolution FEI Quanta 200F field emission scanning electron microscope (FESEM) equipped with an energy dispersive X-ray (EDX) analyzer and transmission electron microscope (TEM, JEOL, TEM2010). The dimension of the layers was measured using SEM micrographs. An X-ray diffraction (XRD) measurement was performed using an Empyrean X-ray diffractometer with Cu Kα radiation (*λ* = 1.54178 Å) operating at 45 kV and 30 mA with a step size of 0.026°, a scanning rate of 0.1°s^−1^, and a 2*θ* range from 10 to 80°. X-ray photoelectron spectroscopy (XPS) analysis was performed using a spectrometer (VG ESCALAB 220i) with an Al Kα excitation source.

### Assessing biocompatibility of the ZrO_2_ surfaces

The cell line MDA-MB-231 containing a constitutively active GFP expression system was utilized for visualization of viable cells. The cells were cultured in T-25 culture flasks using Dulbecco’s modified Eagle’s medium (DMEM) with 1% penstrep (PS) antibiotic and 10% fetal bovine serum (FBS). The cells were maintained in a culture incubator at 37 °C and 5% CO_2_ and when cells reached 80% confluency they were trypsinized, centrifuged, and resuspended in 1 mL of complete DMEM (10% FBS, 1% PS). To each ZrO_2_ surface, 100 uL of the cell suspension was added to the samples and was untouched for 20 minutes followed by addition of 5 mL of complete DMEM (10% FBS, 1% PS) to keep the cells submerged in culture media. The samples were then placed in the culture incubator again for 3 days. On the third day, the cells were fixe by addition of paraformaldehyde and visualization of the cell morphology was performed by fluorescence imaging using a Nikon Eclipse Ti-S inverted microscope with a FITC-LP01 filter set (Semrock Inc.).

## Additional Information

**How to cite this article**: Zalnezhad, E. *et al*. From Zirconium Nanograins to Zirconia Nanoneedles. *Sci. Rep.* **6**, 33282; doi: 10.1038/srep33282 (2016).
